# Pediatric Recurrent Unilateral Trochleitis in Association With Paranasal Sinusitis: A Case Report

**DOI:** 10.7759/cureus.31302

**Published:** 2022-11-09

**Authors:** Ghadah Alnosair, Reem Alqasim

**Affiliations:** 1 Pediatric Ophthalmology and Strabismus, Dammam Medical Complex, Dammam, SAU; 2 Ophthalmology, King Faisal University, Al-Ahsa, SAU

**Keywords:** case report, trochleodynia, pediatric, paranasal sinusitis, trochleitis

## Abstract

Trochleitis is an easily treatable condition; however, it is often misdiagnosed by many clinicians because of its rare incidence. We report the case of a 14-year-old Saudi male patient, known to have type 1 diabetes mellitus (DM) and pansinusitis, who presented to the emergency department with a one-day history of severe right periorbital pain exacerbated by upgaze and supraduction. There was intense point tenderness on palpation over the trochlear region of the orbit with no underlying swelling or redness. Both eyes had a corrected visual acuity of 0.8. Mildly limited elevation (-1) of the right eye was noted. All other extraocular movements were normal in both eyes. Contrast-enhanced CT of the head and orbits showed mild trochlear swelling, inflammation, and pansinusitis. He was treated with a single intratrochlear injection of steroids, which immediately and significantly improved the symptoms. To the best of our knowledge, this is the second case of trochleitis associated with paranasal sinusitis in children. This suggests that a possible, but rare, association between these two conditions may exist in the pediatric population.

## Introduction

Trochleitis is a local inflammation of the trochlea of the superior oblique tendon [1]. It is most often idiopathic and manifests unilaterally [2]. Rarely, trochleitis presents as an ocular manifestation of immunologic and rheumatic disorders such as systemic lupus erythematosis (SLE) and rheumatoid arthritis (RA), which may become sequentially bilateral [3-5]. Patients with trochleitis often complain of periorbital pain localized to the superomedial orbit, radiating to the forehead, and exacerbated by vertical eye movement, especially supraduction [5]. On palpation, tenderness over the trochlea is a very characteristic sign of trochleitis [5]. Some patients experience diplopia, either constantly or transiently [5]. Trochleitis is the result of an inflammatory process; however, patients do not appear to have eyelid swelling or erythema [6]. Although trochleitis is diagnosed mainly based on clinical findings, radiologic evidence of trochlear inflammation on ultrasonography, CT, or MRI usually confirms the diagnosis [7, 8]. Laboratory tests, including routine blood work and serology, are frequently performed to rule out immunologic and rheumatic disorders, as an etiology of this condition [6-8]. Treatment is by local injection of corticosteroids in the trochlear region, which results in remarkable improvement within 48-72 hours in the majority of patients [1,6,9]. Peritrochlear injection of corticosteroids is also of diagnostic value, as the rapid relief of symptoms post-injection can confirm the diagnosis [1].

Although trochleitis is very rare, it is an easily treatable condition. Therefore, it is very important to identify on presentation, as the patient can benefit much from pain relief. However, given the limited number of cases reported in the literature, trochleitis is poorly recognized and often misdiagnosed by many clinicians, especially those who are unfamiliar with this condition. 

Thus, to increase the awareness of clinicians about trochleitis, we present herein a case of recurrent unilateral trochleitis in a teenage boy, known to have type 1 diabetes mellitus (DM) and pansinusitis, who was previously misdiagnosed with optic neuritis.

## Case presentation

History

A 14-year-old Saudi male patient presented to the ED complaining of a one-day history of severe right eye pain. The pain was superonasal of the orbit. It was exacerbated with vertical eye movements, especially upgaze and supraduction. It was also accompanied by vertical diplopia and intense tenderness over the trochlear region of the orbit. There was no history of trauma.

The patient was known to have type 1 DM and was on insulin. He also suffered from chronic pansinusitis. However, he had not been previously diagnosed with rheumatologic and immunologic disorders, including SLE and RA. He had a previous similar episode, for which he also presented to the ED, but was misdiagnosed as having optic neuritis.

On examination

**Figure 1:**
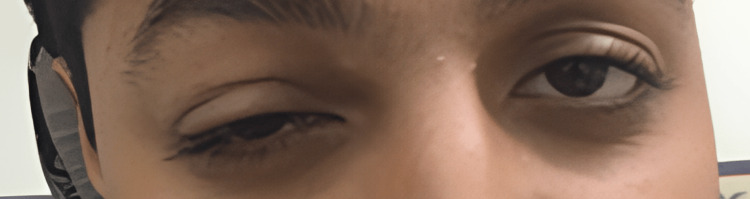
Clinical picture of the patient (pre-injection): note the pseudoptosis due to blepharospasm and pain

On ophthalmological examination, both eyes had a corrected visual acuity of 0.8. Mildly limited elevation (-1) of the right eye was noted. All other extraocular movements were normal in both eyes. Moderate ptosis (3 mm) of the right eye was noted, which was probably due to blepharospasm and severe pain; thus, the patient could not open his right eye completely (Figure 1). In both eyes, the conjunctivae were normal, the corneas were clear, and the anterior chambers were deep and quiet. Both pupils were equal, round, regular, and reactive to light without relative afferent pupillary defect. Fundus examination was unremarkable in both eyes.

Investigations

**Figure 2:**
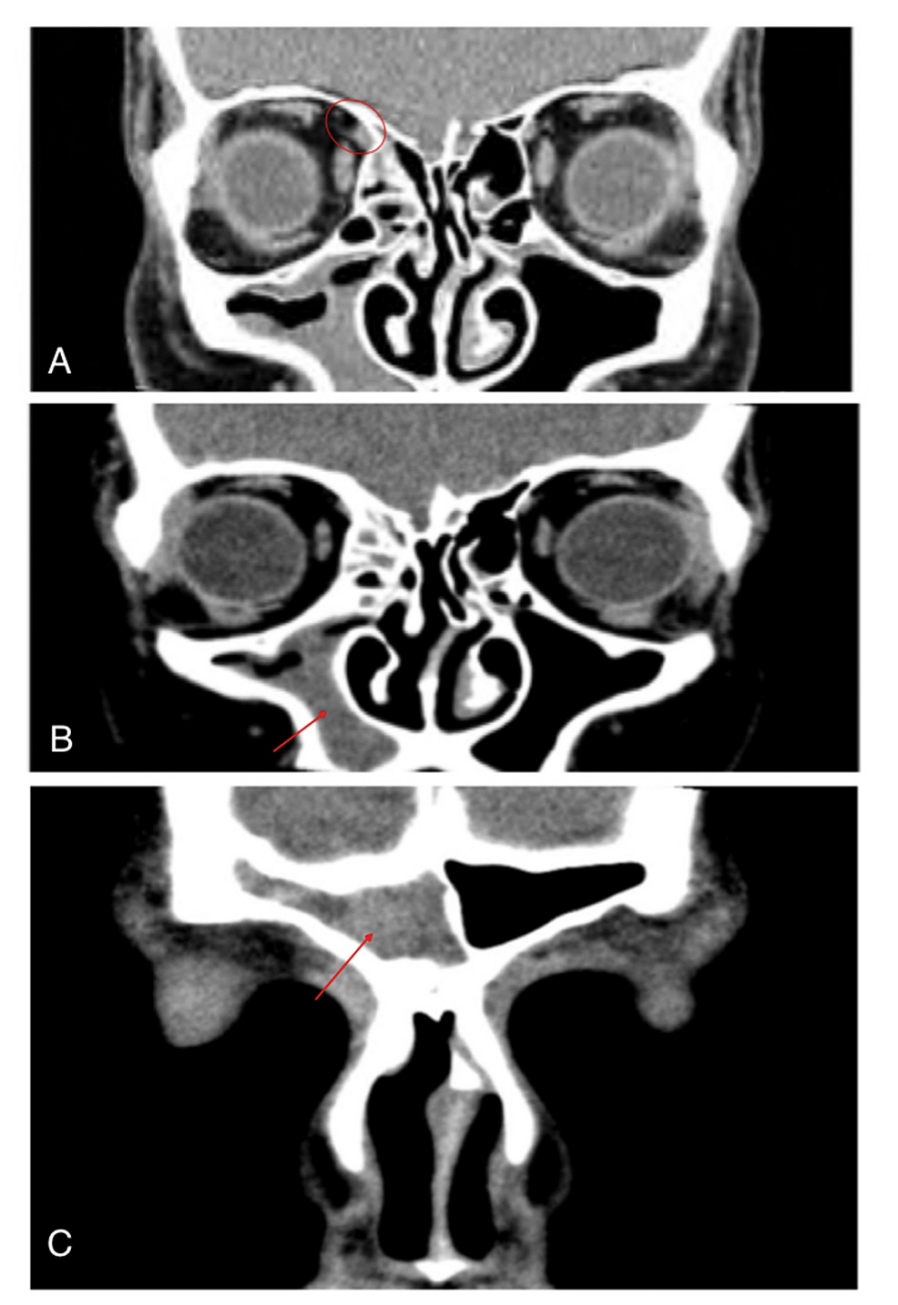
Coronal contrast-enhanced CT scan of orbits and paranasal sinuses showing mild swelling and inflammation of the trochlea of the right eye (A), as well as right-sided maxillary (B) and frontal (C) sinusitis

Contrast-enhanced CT of the head and orbits revealed mild trochlear swelling and inflammation (Figure 2A). In addition, inflammation and mucosal thickening were detected in the right maxillary and frontal sinuses (Figure 2B, 2C), left sphenoid sinus, and both ethmoidal sinuses, indicating a right-sided pan-sinusitis. The patient was referred to an otorhinolaryngologist and started on empirical oral antibiotics. However, the pain was persistent and the tenderness was not resolved, which indicated the need for an intratrochlear injection of steroids. The patient was also referred to a rheumatologist to rule out immunologic and rheumatic disorders such as SLE and RA.

Treatment and follow-up

The patient was diagnosed with trochleitis of the right eye based on the clinical findings and radiological evidence of trochlear inflammation. He was treated with a single steroid injection (0.5 mL of methylprednisolone in combination with 0.5 mL of 2% lidocaine) in the trochlear region of the right eye. A significant improvement was noted immediately after the injection and on follow-up 10 days later. The patient was pain-free for months thereafter.

Assessment of right eye trochleitis post-steroid injection

**Figure 3:**
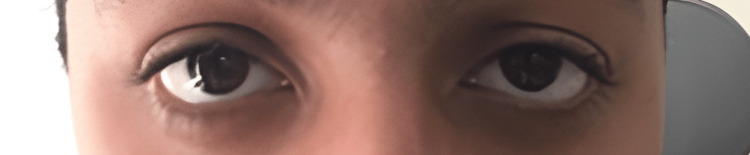
Clinical picture of the patient (post-injection): note how ptosis has disappeared and the patient can open his eye comfortably

The patient only reported mild tenderness over the trochlear region of the orbit. The pain on upgaze, diplopia, and ptosis have resolved. The patient could open his right eye comfortably (Figure 3). His corrected visual acuity of the left and right eyes were 1.0 and 0.9, respectively. In both eyes, the extraocular movements were full, and the anterior chambers were deep and quiet.

## Discussion

Trochleodynia is defined as periorbital pain in the trochlear region that is felt in the superonasal orbit and worsens upon supraduction and palpation [9]. Differential diagnoses include primary trochlear headache, trochlear migraine, inflammatory Brown syndrome, and trochleitis [9-11]. Primary trochlear headache is a headache of unknown etiology that originates from the trochlear region with no evidence of trochlear or systemic inflammation [11]. Trochlear migraine is the coexistence of trochlear pain and migraine headache, with migraine attacks triggered by worsening trochlear pain. The management of trochlear pain helps resolve concurrent migraine attacks, but the opposite is not true [1,9]. Inflammatory Brown syndrome is an acquired restriction in ocular motility, in which the affected eye does not elevate in adduction because of trochlear inflammation [10]. Trochleitis is trochleodynia with trochlear inflammation on radiological imaging such as ultrasonography, CT, or MRI [9].

Trochleitis is mostly idiopathic, and frequently unilateral [2]. Rarely, trochleitis presents bilaterally secondary to immunologic and rheumatic disorders, such as SLE and RA [3-5]. Trochleitis is suspected when the patient complains of trochleodynia and is confirmed when radiological imaging reveals swelling and inflammation in the trochlear region [7, 8]. Systemic workup and laboratory tests should be performed to rule out any underlying systemic inflammatory diseases, especially when trochleitis is bilateral [6-8]. No specific guidelines have been established for the treatment of trochleitis, but the treatment involves the oral administration of non-steroidal anti-inflammatory drugs (NSAIDs), oral administration of steroids, or topical injections of steroids [5,8,11]. Isolated trochlear pain may be relieved with oral NSAIDs, but the response might not be adequate if the pain is associated with diplopia and restricted ocular motility [8]. However, in the majority of cases, topical injections of steroids are more effective in providing a rapid and complete resolution of symptoms [1,6,9].

As far as we have known, trochleitis in association with paranasal sinus inflammation has only been reported once in a pediatric patient (aged eight years) [12]. One case of a post-surgical inflammatory Brown syndrome associated with trochleitis following a frontal sinus surgery may also be relevant [13].

To the best of our knowledge, this is the second case of trochleitis associated with paranasal sinusitis in a pediatric patient (aged 14 years). One suggested explanation for this association is the anatomical proximity between the paranasal sinuses and the orbit along with its contents, including the trochlea of the superior oblique tendon [12].

In the present case, the patient’s clinical findings were very typical of trochleitis. Nevertheless, he was first misdiagnosed with optic neuritis because he was known to be diabetic. This has proven that trochleitis is poorly recognized by clinicians, as they rarely encountered this condition and hence are unfamiliar with it.

Fortunately, when the patient presented to the ED with the same complaint for the second time, the pediatric ophthalmologist, who was on call, managed to diagnose unilateral trochleitis as the underlying cause of the recurrent symptoms. Early intervention with a single intratrochlear injection of steroids helped resolved his symptoms.

## Conclusions

By reporting this case, we hope to increase the awareness of trochleitis among clinicians and thus prevent any delay in its diagnosis and treatment, which is simple and effective. This case also highlights the rare but possible association between trochleitis and paranasal sinusitis in children.
